# Examining the Effects of Dasatinib, Sorafenib, and Nilotinib on Vascular Smooth Muscle Cells: Insights into Proliferation, Migration, and Gene Expression Dynamics

**DOI:** 10.3390/diseases11040147

**Published:** 2023-10-23

**Authors:** Khalid Alhazzani, Abdullah Almangour, Abdulaziz Alsalem, Mohammed Alqinyah, Abdullah S. Alhamed, Hussain N. Alhamami, Ahmed Z. Alanazi

**Affiliations:** Department of Pharmacology and Toxicology, College of Pharmacy, King Saud University, Riyadh 11451, Saudi Arabia

**Keywords:** vascular smooth muscle, dasatinib, sorafenib, nilotinib

## Abstract

Background: Dasatinib, nilotinib, and sorafenib are clinically proven tyrosine kinase inhibitors (TKIs) used for the treatment of leukemia and hepatocellular carcinoma. However, there is a growing concern regarding cardiotoxicity associated with their use. The impact of these TKIs on vascular smooth muscle cells (VSMCs) remains unexplored. This study aims to investigate the effects of TKIs on VSMC proliferation and migration, as well as to elucidate the underlying mechanisms involving inflammatory and apoptotic pathways. Methods: VSMCs were extracted from albino rats and cultured in vitro. The cells were divided into four experimental groups: control, dasatinib, sorafenib, and nilotinib. The MTT assay was employed to assess the cytotoxic effects of TKIs on VSMCs. A scratch assay was conducted to evaluate the inhibitory potential of TKIs on VSMC migration. Flow cytometry analysis was used to detect apoptotic cells. Real-Time PCR expression was utilized to determine the differential gene expression of apoptotic and inflammatory markers. Results: Dasatinib, nilotinib, and sorafenib demonstrated significant inhibitory effects on VSMC viability and migration at low concentrations (<1 µmol/L, *p* < 0.05). Furthermore, gene expression analysis revealed up-regulation of inflammatory biomarkers (TNF-α, IL-6, and IL-1β) and apoptotic markers (P53, BAX), along with down-regulation of the anti-apoptotic biomarker BCL-2 in response to all TKIs. Conclusions: This study demonstrates that dasatinib, nilotinib, and sorafenib inhibit VSMC proliferation and migration, suggesting their potential to induce vascular injury and remodeling by activating inflammation and apoptosis pathways. These findings highlight the need for further investigation into the cardiotoxic effects of these TKIs and the development of strategies to mitigate their adverse vascular effects.

## 1. Introduction

In the past decades, the discovery of tyrosine kinase inhibitors (TKIs) has tremendously transformed cancer treatment [1]. Due to these drugs, several malignant tumors that were previously fatal are now easier to control. However, the use of TKIs are associated with significant cardiovascular toxicity that is clinically manifested by hypertension, systolic dysfunction, and heart failure [2,3]. In addition, patients who receive TKI live longer, which further increases the risk of cardiovascular complications leading to cardiac remodeling, manifested by morphological changes in the heart’s size and shape in response to disease or damage.

**Figure 1:**
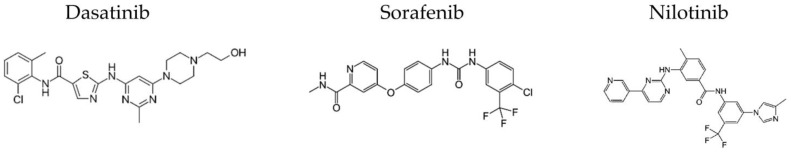
Chemical structures of dasatinib, sorafenib, and nilotinib. It illustrates the molecular structures of dasatinib (C_22_H_26_ClN_7_O_2_S), sorafenib (C_21_H_16_ClF_3_N_4_O_3_), and nilotinib (C_28_H_22_F_3_N_7_O).

Several TKIs have been approved to treat cancer, including sorafenib, dasatinib, and nilotinib (Figure 1). Dasatinib is primarily indicated for the treatment of chronic myeloid leukemia (CML) and acute lymphoblastic leukemia (ALL), especially in cases where the disease shows resistance to imatinib, the first-generation TKI. It functions by inhibiting the BCR-ABL tyrosine kinase, a protein that encourages the growth of leukemia cells. Similarly, nilotinib is used for the treatment of chronic phase and accelerated phase Philadelphia chromosome positive (Ph+) CML in adults. Similar to dasatinib, nilotinib also blocks the BCR-ABL tyrosine kinase, thereby inhibiting the growth of leukemia cells. In addition to these, sorafenib is another tyrosine kinase inhibitor that blocks the RAF/MEK/ERK pathway. It is primarily employed in the treatment of advanced renal cell carcinoma, hepatocellular carcinoma, and thyroid carcinoma [1]. Despite their clinical benefits, these drugs are often poorly selective and inhibit many kinases that are not involved in malignant tumors. These drugs may have an effect on vascular smooth muscle cells (VSMCs) that line all arteries and veins of the human body, which is consist of long and thin cells with one central nucleus, and many protein fibers from actin and myosin, which makes them play an important role in maintaining blood pressure and oxygen flow through the body and controlling it [4].

In a clinical trial, patients treated with sorafenib showed increased incidents of cardiac ischemia compared with the placebo group (3 vs. <1%, *p* = 0.01) [5]. In addition, sorafenib cardiotoxicity is still manifested after discontinuation of therapy for 3 months as reported by Uraizee et al. [6]. There have also been four published case reports representing the occurrence of acute coronary artery disease in patients on sorafenib therapy [7,8].

On the other hand, dasatinib is recommended to be avoided during pregnancy due to a case-report study in 2017 showing a cardiovascular defect in an infant born to a woman on dasatinib treatment [9]. Also, another study concluded that with a variability in the effect of cardiotoxicity of the tyrosine kinase inhibitors class and variable adverse effects associated with each agent, the result showed elevated blood pressure to be most common with sorafenib, fluid retention to be most common in dasatinib treatment, and lowered LVEF in lapatinib [10]. In addition to that, there is a retrospective cohort study that monitors 179 patients using nilotinib who have chronic myeloid leukemia to detect any development or onset of peripheral artery disease (PAD), and they found 11 (6.15%) patients with severe and previously unrecognized PAD that required invasive therapy. After that, they recommend monitoring any patients who are at risk for PAD [11].

In fact, there are no studies evaluating the direct relationship between the toxicity of vascular smooth muscle cells (VSMCs) and the protein kinase inhibitors (sorafenib, dasatinib and nilotinib). However, there are several studies that have shown an indirect relationship between VSMCs and sorafenib. Accordingly, a case-report study published in 2014 showed that the patient treated with sorafenib developed hypertension and an aortic dilation detected by abdominal computerized tomography and echocardiography. Which was well controlled with β-blocker medication [12]. Also, in this observational, single-center study of 74 patients using either sorafenib or sunitinib, 33.8% had a cardiac event, 40.5% had ECG changes, 18% were symptomatic, and 9.4% of these patients were seriously compromised and required intensive care admission, so careful cardiovascular monitoring should be considered [13]. To the best of our knowledge, no study has been conducted to investigate the effects of TKIs on inducing cardiovascular remodeling using VSMCs. We hypothesized that TKIS will induce inflammation gene expression on vascular smooth muscle cells, leading to detrimental effects of vascular remodeling and toxicity.

## 2. Materials and Methods

### 2.1. Materials

Sorafenib, dasatinib, and nilotinib were purchased from MedChemExpress (MCE) (Monmouth Junction, NJ, USA). Material used in tissue culture, such as 100-mm, 6-well, and T-75 culture plates, were obtained from Fisher Scientific (Milford, MA, USA). The methyl thiazolyl tetrazolium (MTT) assay was procured from UFC Biotechnology (Buffalo, NY, USA). Flow cytometry kits using propidium iodide (PI) and annexin V staining were obtained from ThermoFisher (Waltham, MA, USA). Primers of p53, BAX, BCL-2, TNF-α, IL-6, IL-1β, and GAPDH as an internal housekeeping gene were procured from Integrated DNA Technologies (Leuven, Belgium).

### 2.2. Extraction of Vascular Smooth Muscles

Albino rats (male, 40 days old, weighing 120 g) were obtained from the animal care facility located at the Pharmacy College, King Saud University. Animal handling, maintenance, and surgical procedures were performed according to the rules and guidelines approved by the Animal Care & Use Committee (KSU-SE-23–28). To obtain a primary culture, VSMCs were extracted from the thoracic aorta using the explant technique, as previously described [14]. Briefly, the extracted aorta was placed in a 100 mm cell culture plate containing sterile phosphate buffer to wash out blood and unwanted tissues. Then, the thoracic aorta was excised into strips and placed into culture plates prefilled with DMEM-F12 media to allow VSMCs to grow. Once the VSMCs had grown sufficiently, cells were transferred into larger cell culture flasks (T-75) to obtain ample cells in order to explore and probe the effects of TKIs on these cells.

### 2.3. Cell Culture and Experimental Design

VSMCs were extracted from albino rats and allowed to grow in vitro in DMEM-F12 media supplemented with 10% fetal bovine serum, 100 units/mL streptomycin, and 100 µg/mL penicillin. The VSMCs were maintained in a humidified incubator with 5% CO_2_ at 37 °C throughout the experiment. The day before the treatment challenge, VSMCs were seeded into 6-well plates to allow cellular adhesion to the bottom of the wells. On the following day, the wells were divided into four main groups that were treated as follows: Group I: served as the control and was treated with vehicle for 24 h. Group II: was treated with increasing concentrations of dasatinib for 24 h. Group III: was treated with increasing concentrations of sorafenib for 24 h. Group IV: was treated with increasing concentrations of nilotinib for 24 h.

### 2.4. Cell Viability Assay

The methyl thiazolyl tetrazolium (MTT) assay was performed to assess cellular viability of VSMCs, following a previously described method [15]. Briefly, VSMCs were plated in a 96-well cell culture plate and treated with different concentrations of dasatinib, sorafenib, and nilotinib (0.05, 0.1, 0.25, 0.5, 1 µmol/L), while the control group was treated with the vehicle (5% DMSO). After 24 h of drug challenge, the supernatant was removed and replaced with MTT (5 mg/mL), which was then incubated for 3 h to allow formazan crystals to form. Subsequently, DMSO was added, and the plate was subjected to gentle shaking to dissolve the crystals in order to read the absorbance at 570 mm by a plate reader.

### 2.5. Scratch Assay

The scratch assay was deployed to evaluate the inhibitory effects of TKIs on the migration of VSMCs [16]. Briefly, VSMCs were cultured in 6-well cell culture plates and incubated in a humidified incubator to establish cell monolayers. Then, a sterile 200 μL tip was used to scratch the cell monolayers, creating a single straight-line wound area. Cells were then photographed twice: at 0 h (immediately post-scratch and before treatment) and at 24 h (after being treated with indicated drugs for 24 h). Images were obtained using an EVOS XL Core Microscope (ThermoFisher, Waltham, MA, USA). 

### 2.6. Flow Cytometry Analysis of Apoptosis

Apoptotic cells were detected using Annexin V/propidium iodide kits for flow cytometry, as described [15]. Briefly, cells were treated for 24 h with dasatinib, sorafenib, and nilotinib. Then, VMCSs were washed at least three times with ice-cold PBS in order to be harvested using 25% trypsin, followed by centrifugation at 300 g for 5 min. VSMCs were resuspended in annexin V and PI binding buffer with gentle shaking in a dark room for 15 min. Finally, VSMCs were subjected to flow cytometry analysis using a Beckman Coulter Cytomics FC 500 (Pasadena, CA, USA). Data were obtained using the manufacturer’s software.

### 2.7. RNA Isolation and Gene Expression

VSMCs were cultured in 12-well cell culture plates and treated with different concentrations of dasatinib, sorafenib, and nilotinib for 24 h. Then, RNA was extracted using the TRIzol extraction method. The purity and concentration of the extracted RNA were checked using NanoDrop^TM^ 8000 spectrophotometry (ThermoFisher, Waltham, MA, USA). If the OD 260/280 ratio was greater than 1.8, the extracted RNA was considered acceptable, and the process of reverse transcription began using the MCE^®^ Reverse Transcription Kit (MedChemExpress, Monmouth Junction, NJ, USA) to obtain single-stranded complementary DNA. Next, a PCR reaction was prepared by mixing cDNA, forward and reverse primers of the gene of interest, and SYBR Green dye. The primer sequences for p53, IL-6, IL-1β, BCL-2, BAX, TNF-α, and GAPDH is listed in Table 1. Then, the PCR reaction was loaded into a Bio-Rad RT-PCR machine (Hercules, CA, USA). The difference in gene expression was estimated using the 2^−△△Ct^ method, in which GAPDH was used as an endogenous control gene (housekeeping gene). 

### 2.8. Statistical Analysis & Graphs

All data presented in graphs were expressed as mean ± standard deviation (S.D). All statistical analysis as well as graphs were generated using GraphPad Prism (version 9.5.0). To analyze statistical significance, One-way ANOVA was used to detect statistical significance among different experimental groups, followed by Dunnett’s or Tukey’s multiple comparison tests as post-hoc tests to determine statistical significance between experimental groups. A statistically significant result is achieved if the *p*-value is less than or equal to 0.05 (*p* ≤ 0.05).

## 3. Results

### 3.1. Cell Viability

**Figure 2:**
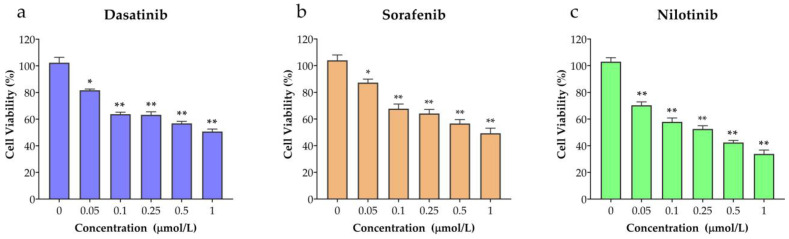
The inhibitory effects of (**a**) dasatinib, (**b**) sorafenib, (**c**) nilotinib on VSMCs viability. Cell growth was assessed by the MTT assay after treatment with dasatinib, sorafenib, and nilotinib at concentration ranges (0.05, 0.1, 0.25, 0.5, 1 µmol/L). * *p* ≤ 0.05; ** *p* ≤ 0.05 compared to the control group.

To determine the effects of dasatinib, sorafenib, and nilotinib on the viability of VSMCs, cells were subjected to increasing concentrations (0.05, 0.1, 0.25, 0.5, 1 µmol/L) of these TKIs for 24 h and assessed using the MTT assay. As shown in Figure 2a–c, the viability of VSMCs was significantly reduced in a concentration-dependent manner. Treatment with nilotinib showed markedly lower cell viability than dasatinib and sorafenib.

### 3.2. Cell Apoptosis

**Figure 3:**
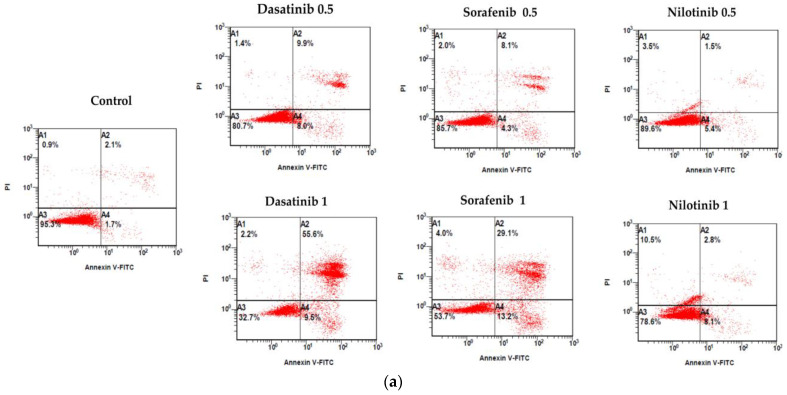

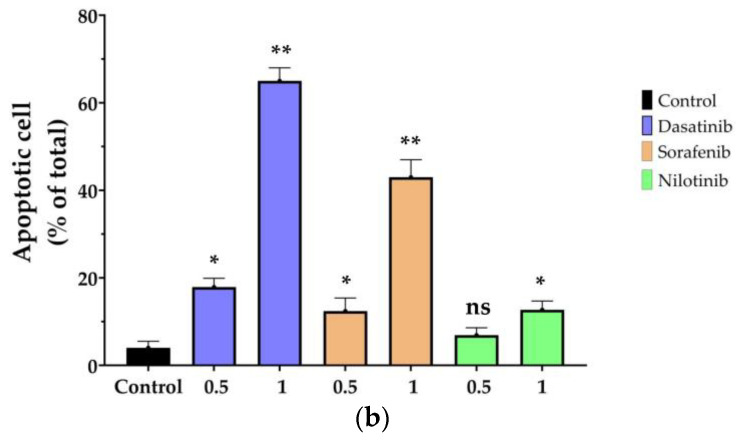
Annexin V plus PI staining assay of VSMCs treated with dasatinib, sorafenib, nilotinib. (**a**) Flow cytometric analysis of cells that were challenged with dasatinib (0.5 µmol/L, 1 µmol/L), sorafenib (0.5 µmol/L, 1 µmol/L), nilotinib (0.5 µmol/L, 1 µmol/L) for 24 h. (**b**) Statistical graph of Annexin V plus PI stained VMSCs. ns indicates *p* > 0.05; * *p* ≤ 0.05; ** *p* ≤ 0.01 compared to the control group.

As mentioned previously, dasatinib, sorafenib, and nilotinib exhibited inhibitory effect on VSMCS viability. Therefore, we investigated the impact of these TKIs on the induction of apoptosis in VSMCs using flow cytometry. In these experiments, VSMCs were treated for 24 h with 0.5 µmol/L and 1 µmol/L concentrations of dasatinib, sorafenib, and nilotinib and then probed with annexin V and propidium iodide (PI) staining. As shown in Figure 3a,b, the results showed differential effects of TKIs on VSMCs cellular apoptosis, in which dasatinib showed the strongest effects towards induction of apoptosis (65%) followed by sorafenib (42%), whereas nilotinib exhibited the lowest effects (11%).

### 3.3. Cellular Migration Assay

**Figure 4:**
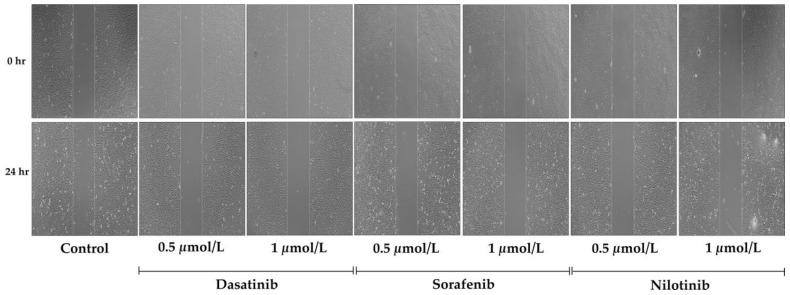
Time-lapse microscopy images of VSMCs migration. The migration was measured by scratch wound healing assay of untreated (control) or treated cells with dasatinib (0.5 µmol/L, 1 µmol/L), sorafenib (0.5 µmol/L, 1 µmol/L), and nilotinib (0.5 µmol/L, 1 µmol/L) at 0, 24 h. The dotted line defines the area lacking migratory cells. Magnification ×2000.

A scratch assay was conducted to explore the potential inhibitory effects of TKIs on the migration of VSMCs. Immediately after the scratch (0 h), VSMCs were treated with varying concentrations of dasatinib (0.5 and 1 µmol/L), sorafenib (0.5 and 1 µmol/L), and nilotinib (0.5 and 1 µmol/L) for a duration of 24 h. Our observations clearly indicated a significant decrease in VSMC migration following treatment with all three TKIs. This was evidenced by the lack of closure in the wound area, which typically signifies cell migration. This suggests that these TKIs may play a role in inhibiting VSMC migration, a key process in vascular remodeling. These findings are visually represented in Figure 4.

### 3.4. Apoptosis-Related Gene Expression

**Figure 5:**
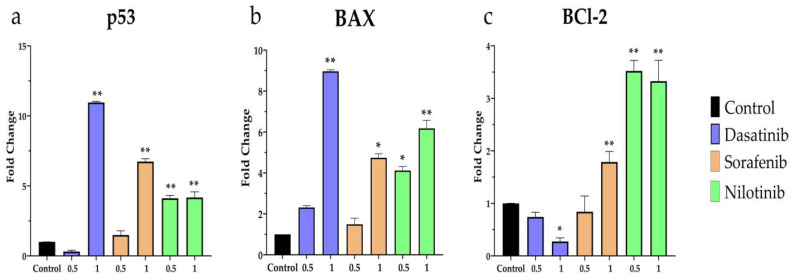
RT-PCR analysis of (**a**) p53, (**b**) BAX, (**c**) BCL-2 mRNA expression in VSMCs after treatment with dasatinib (0.5 µmol/L, 1 µmol/L), sorafenib (0.5 µmol/L, 1 µmol/L), and nilotinib (0.5 µmol/L 1 µmol/L) for 24 h. * *p* ≤ 0.05; ** *p* ≤ 0.01 compared to the control group.

Real-time PCR was executed to investigate the changes in the gene expression levels of apoptotic-related genes such as p53, BAX, and BCL-2 after treatment with TKIs. In these sets of experiments, VSMCS were treated with dasatinib (0.5 and 1 µmol/L), sorafenib (0.5 and 1 µmol/L), and nilotinib (0.5 and 1 µmol/L) for 24 h. As shown in Figure 5a, p53 expression was significantly upregulated upon treatment with 1 µmol/L of either dasatinib or sorafenib, whereas nilotinib showed higher expression of p53 at both concentrations 0.5 and 1 µmol/L. Similarly, the pro-apoptotic gene expression of BAX (Figure 5b) was significantly increased in dasatinib (1 µmol/L), sorafenib (1 µmol/L), and nilotinib (0.5 and 1 µmol/L). On the other hand, the anti-apoptotic gene expression of BCL-2 was significantly downregulated after dasatinib treatment, whereas sorafenib and nilotinib showed upregulation of BCL-2.

### 3.5. Inflammation-Related Gene Expression

**Figure 6:**
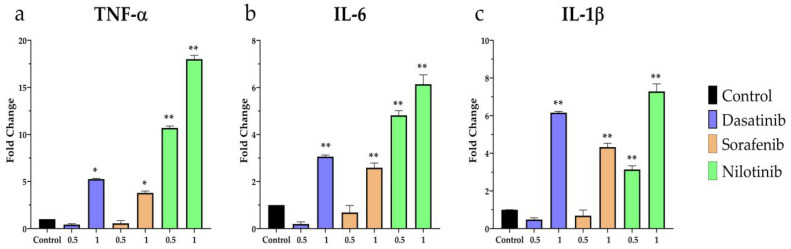
RT-PCR analysis of (**a**) TNF-α, (**b**) IL-6, (**c**) IL-1β mRNA expression in VSMCs after treatment with dasatinib, sorafenib, nilotinib for 24 h at 0.5 and 1 µmol/L concentrations. * *p* ≤ 0.05, ** *p* ≤ 0.01 compared to the control group.

Similarly, the expression of inflammatory-related genes TNF-α, IL-6, and IL-1β was assessed using quantitative real-time PCR. As depicted in Figure 6a, TNF-α expression was significantly upregulated upon treatment with nilotinib at both concentrations 0.5 and 1 µmol/L whereas dasatinib and sorafenib exhibited higher expression only at 1 µmol/L. likewise, the expression of IL-6 and IL-1β was upregulated following treatment with dasatinib (1 µmol/L), sorafenib (1 µmol/L), and nilotinib (0.5 and 1 µmol/L) as shown in Figure 6b,c.

## 4. Discussion

The human cardiovascular system, an intricate network of blood vessels and the heart, relies extensively on the functional attributes of VSMCs for optimal performance. These cells are crucial regulators of vascular tone and significantly contribute to vascular remodeling, a phenomenon often linked to the pathogenesis of cardiovascular disorders, including hypertension [17]. Thus, any factors interacting with and influencing VSMC behavior, such as TKIs like dasatinib, sorafenib, and nilotinib, are of paramount interest to clinicians and researchers in understanding and mitigating cardiovascular diseases. As our results demonstrate, the TKIs can induce a state of cellular dysregulation in VSMCs, potentially leading to pathological vascular changes characteristic of cardiovascular diseases. This understanding of the specific cellular pathways affected by these agents is crucial as TKIs emerge as potent tools in cancer therapeutics.

TKIs have been shown to significantly impede cell growth. The mechanism involves the inhibition of tyrosine kinases, which participate in cell growth signal transduction pathways. Imatinib, a TKI initially developed to treat chronic myeloid leukemia (CML), inhibits the BCR-ABL tyrosine kinase, a fusion protein constitutively promoting cell proliferation. Druker et al. and Deininger et al. [18,19] demonstrated Imatinib’s effectiveness in CML treatment by significantly reducing cell growth. Research also indicates that TKIs influence cell migration, mainly via the inhibition of growth factor receptors that stimulate cell movement. Sorafenib, a multi-kinase inhibitor, was shown to inhibit cell migration in hepatocellular carcinoma by blocking the Raf/MEK/ERK pathway [20]. Sunitinib, another multi-kinase inhibitor, displayed similar effects on cell migration in renal cell carcinoma [21].

In addition to suppressing growth and migration, TKIs can induce apoptosis in cancer cells. Imatinib, for instance, promotes apoptosis in BCR-ABL positive cells. Similarly, Lapatinib, a dual inhibitor for EGFR/HER2 receptors, has been shown to induce apoptosis in HER2-overexpressing breast cancer cells [18,22]. Our experimental results align with the findings of previous studies that demonstrate the impact of TKIs on cell growth, migration, and apoptosis. We observed that TKIs trigger a cascade of proinflammatory and pro-apoptotic mediators with a significant detrimental impact on VSMC survival and motility, similar to the outcomes of previous studies [23].

Interestingly, we also observed significant expression patterns of critical genes involved in several cellular processes. These include P53, BAX, BCL-2, TNF-α, IL-6, and IL-1β, which are renowned for their roles in cell growth regulation, inflammation, and apoptosis. The findings are also consistent with what has been described previously [24]. They reported the alteration of these genes’ expression following TKI treatment, implying a common mechanism of TKI action on VSMC. In fact, the roles of TNF-α and IL-6 in VSMCs have been the subject of extensive research due to their significant implications for cardiovascular diseases such as atherosclerosis, restenosis, and hypertension. Both of these cytokines have been implicated in proliferation, migration, and inflammatory responses of VSMCs [25,26,27].

A study published by Boring et al. suggested that TNF-α can stimulate the proliferation and migration of VSMCs, which are key steps in the formation of atherosclerotic plaques and restenosis. This study indicated the possible role of TNF-α as a therapeutic target [28]. Bennett et al. demonstrated that TNF-α also promotes the expression of adhesion molecules in VSMCs, thereby promoting the adhesion and infiltration of leukocytes. This is a key process in the inflammation observed in atherosclerosis [29].

The role of IL-6 in VSMCs has also been extensively studied. Schieffer et al. (2000) demonstrated that IL-6 is involved in the proliferation and migration of VSMCs, similar to TNF-α. IL-6 has also been found to be a major regulator of inflammation in VSMCs. A study by Romano et al. (1997) showed that IL-6 can induce the production of acute-phase proteins, which are involved in the inflammation associated with atherosclerosis [30,31].

Interestingly, some studies have also pointed out that both TNF-α and IL-6 may have a dual role in VSMCs. While they are generally considered to be pro-atherogenic, under some conditions, they may also exert protective effects. For example, a study by Grote et al. (2005) suggested that TNF-α can induce apoptosis in VSMCs, which may contribute to the resolution of inflammation and plaque stabilization [32]. So, the roles of TNF-α and IL-6 in VSMCs are complex, and their implications for cardiovascular diseases are significant. Both cytokines have been implicated in key processes such as proliferation, migration, and inflammation.

The clinical implications of TKI-induced cardiotoxicity could be profound, given their increasing usage in cancer treatment. The balance between the therapeutic benefits of TKIs and the potential risk of cardiovascular complications is a complex challenge for clinicians and oncologists. This echoes the findings of Delombaerde et al., who highlighted the need for comprehensive cardiovascular monitoring in patients undergoing TKI therapy [33].

Despite these significant findings, our study underlines the need for extensive research into the exact cellular and molecular mechanisms underpinning the effects of TKIs on VSMCs and vascular remodeling. While our findings provide valuable insights, the exact interplay between these inhibitors, VSMCs, and the cardiovascular system as a whole remains largely uncharted territory that requires further exploration. In this context, we acknowledge two notable limitations in our study of vascular toxicity. First, the study did not include endothelial cells, which are crucial in maintaining vascular integrity, regulating blood flow, and mediating tissue interactions. The inclusion of endothelial cells in future studies will broaden our understanding of the specific effects of dasatinib, nilotinib, and sorafenib on endothelial function and potential toxicity. Second, the study did not explore the impact of these TKIs on cardiomyocytes, which are essential for proper cardiac function. The lack of cardiomyocytes has limited our understanding of potential cardiotoxic effects associated with the use of these drugs.

Future studies should investigate the interactions between TKIs and other critical components of the cardiovascular system. Given the systemic nature of cardiovascular diseases, it is plausible that TKIs could have broader effects beyond their interaction with VSMCs. Such investigations could revolutionize the therapeutic management of cancer patients with pre-existing cardiovascular conditions or those at risk due to TKI treatment.

So, our research makes a significant contribution towards understanding the complex relationship between TKIs and VSMCs. However, a comprehensive understanding of the issue and thereby mitigating potential harm necessitates a multidimensional, more in-depth approach, embracing broader aspects of the cardiovascular system. This work could pave the way for groundbreaking therapeutic strategies and hold the key to a delicate balance between the benefits and potential risks of TKIs in cancer treatment.

## 5. Conclusions

Our study showed that dasatinib, nilotinib, and sorafenib are able to halt VSMCs proliferation and migration, which may give evidence of their propensity to inflict vascular injury and damage. Our results also suggested that these TKIs might trigger VSMCs inflammation and apoptosis, which play a central role in their toxicity towards VSMCs, a critical component of cardiovascular system.

## Figures and Tables

**Table 1:** Primer Sequences for Apoptosis-Related and Inflammation-Related Genes.

Gene	5′-Forward Sequence-3′	5′-Reverse Sequence-3′
**P53**	ACATGACTGAGGTCGTGAGA	GATTTCCTTCCACCCGGATAAG
**BAX**	GATGGCCTCCTTTCCTACTTC	CTTCTTCCAGATGGTGAGTGAG
**BCL-2**	GGAGGATTGTGGCCT TCT TT	GTGAGCTGAGTGGAGAAGAAG
**TNF-α**	TCCTTCAGACACCCTCAACC	AGGCCCCAGTTTGAATTCTT
**IL-6**	TCTGGAGTTCCGTTTCTACCTGG	CATAGCACACTAGGTTTGCCGAG
**IL-1β**	CTATGGCAACTG TCCCTGAA	GGCTTGGAAGCAATCCTTAATC
**GAPDH**	GTATTGGGCGCCTGGTCACC	CGCTCCTGGAAGATGGTGATGG

## Data Availability

All data supporting the findings of this study are contained within the manuscript.
